# An analysis of neutrophil-to-lymphocyte ratios and monocyte-to-lymphocyte ratios with six-month prognosis after cerebral contusions

**DOI:** 10.3389/fimmu.2024.1336862

**Published:** 2024-03-12

**Authors:** Dangui Zhang, Dongzhou Zhuang, Tian Li, Xueer Liu, Zelin Zhang, Lihong Zhu, Fei Tian, Xiaoxuan Chen, Kangsheng Li, Weiqiang Chen, Jiangtao Sheng

**Affiliations:** ^1^ Research Center of Translational Medicine, Second Affiliated Hospital of Shantou University Medical College, Shantou, Guangdong, China; ^2^ Department of Neurosurgery, First Affiliated Hospital of Shantou University Medical College, Shantou, Guangdong, China; ^3^ Department of Microbiology and Immunology and Guangdong Provincial Key Laboratory of Infectious Disease and Molecular Immunopathology, Shantou University Medical College, Shantou, Guangdong, China; ^4^ Department of Neurosurgery, Second Affiliated Hospital of Shantou University Medical College, Shantou, Guangdong, China

**Keywords:** cerebral contusion, traumatic brain injury, neutrophil-to-lymphocyte ratio, monocyte-to-lymphocyte ratio, inflammatory index, long-term unfavorable prognosis

## Abstract

**Background and purpose:**

Neutrophil-to-lymphocyte ratio (NLR) and monocyte-to-lymphocyte ratio (MLR) have been identified as potential prognostic markers in various conditions, including cancer, cardiovascular disease, and stroke. This study aims to investigate the dynamic changes of NLR and MLR following cerebral contusion and their associations with six-month outcomes.

**Methods:**

Retrospective data were collected from January 2016 to April 2020, including patients diagnosed with cerebral contusion and discharged from two teaching-oriented tertiary hospitals in Southern China. Patient demographics, clinical manifestations, laboratory test results (neutrophil, monocyte, and lymphocyte counts) obtained at admission, 24 hours, and one week after cerebral contusion, as well as outcomes, were analyzed. An unfavorable outcome was defined as a Glasgow Outcome Score (GOS) of 0-3 at six months. Logistic regression analysis was performed to identify independent predictors of prognosis, while receiver characteristic curve analysis was used to determine the optimal cutoff values for NLR and MLR.

**Results:**

A total of 552 patients (mean age 47.40, SD 17.09) were included, with 73.19% being male. Higher NLR at one-week post-cerebral contusion (adjusted OR = 4.19, 95%CI, 1.16 - 15.16, *P* = 0.029) and higher MLR at admission and at 24 h (5.80, 1.40 - 24.02, *P* = 0.015; 9.06, 1.45 - 56.54, *P* = 0.018, respectively) were significantly associated with a 6-month unfavorable prognosis after adjustment for other risk factors by multiple logistic regression. The NLR at admission and 24 hours, as well as the MLR at one week, were not significant predictors for a 6-month unfavorable prognosis. Based on receiver operating characteristic curve analysis, the optimal thresholds of NLR at 1 week and MLR at admission after cerebral contusion that best discriminated a unfavorable outcome at 6-month were 6.39 (81.60% sensitivity and 70.73% specificity) and 0.76 (55.47% sensitivity and 78.26% specificity), respectively.

**Conclusion:**

NLR measured one week after cerebral contusion and MLR measured at admission may serve as predictive markers for a 6-month unfavorable prognosis. These ratios hold potential as parameters for risk stratification in patients with cerebral contusion, complementing established biomarkers in diagnosis and treatment. However, further prospective studies with larger cohorts are needed to validate these findings.

## Introduction

Globally, traumatic brain injury (TBI) remains a primary factor in traumatic deaths and is expected to exceed many other disorders as a leading cause of death and disability by 2020 (1). Severe TBI has a rate of around 13 cases per 10,0000 people in both China and other countries, with high mortality rates of 30 to 40% and the potential to cause disability in up to 60% of cases (2, 3). TBI includes several types of insults to the brain. One of the most severe damage mechanisms is hemorrhagic cerebral contusion. More than half of patients with severe TBI experience a cerebral contusion (4). Among adults over 60 with TBI, cerebral contusions are found in around 18% of cases (5). In pediatric TBI, brain contusion (55.9%) is also frequently observed (6). These contusions not only cause loss of function but also pose a significant threat to healthy brain tissue due to the toxic nature of blood. Cerebral lesions occur shortly after the head impact, and contusions can progress and involve other areas of the brain. TBI associated with cerebral contusions apparently increases the risks for disability and death in TBI patients (7).

Noncontrast computed tomography (CT) (NCCT) scans are commonly employed for the initial diagnosis of segmental brain contusions. Once the head CT scan is obtained, management is decided for patients with cerebral contusion (8). However, discrepancies in the interpretation of CT results are commonly reported clinically, especially in emergency departments (9). Thus, seeking CT images with interpretation for more than one time-point could increase the accuracy and was applied in this study to investigate the association between NLR or MLR and hematoma volume in cerebral contusion. On the other hand, an admission warning strategy which comprises 10 indicators, including baseline characteristics (age, GCS score, pupillary reactivity and hypotension), imaging indicators (midline shift, intracerebral hematoma, traumatic subarachnoid hematoma, and basal cistern), and laboratory indicators (glucose level and monocyte count), was developed and validated to predict the outcome of TBI patients. This warning system was proved to have a good discrimination with short-term outcomes (10).

When treating patients with severe brain contusion, it is crucial to monitor internal pressure (ICP), especially when their Glasgow Coma Scale (GCS) is lower than 8. Microvascular decompression is a recognized treatment option for releasing ICP (11). In addition to traditional treatment methods, cutting-edge treatment technologies are also being developed and tested. The use of mesenchymal stem cell-derived exosomal treatment has been proven to have a positive effect on preventing neuro injury and could be a potential therapeutic strategy for cerebral contusion (12).

Despite significant advancements in treatment and care, the mortality rate for TBI remains at 30–50% (13). Therefore, identify specific blood markers associated with the prognosis of TBI is thought to be important and helpful in managing the prognosis management of cerebral contusion (3, 7, 8).

Not only do CT imaging indicators help in diagnosing brain contusions, but they are also a key tool for predicting the prognosis of patients with brain contusions. Postoperative CT score after decompressive craniectomy (DC) is a valuable predictor for early mortality in severe TBI patients after DC (14).

Additionally, specialized serological markers can be created to predict the prognosis of individuals with brain contusions. For example, serum troponin has been reported as an independent predictor for level I trauma prognosis (14). The neutrophil-lymphocyte ratio (NLR) and monocyte-lymphocyte ratio (MLR) are the two inflammatory biomarkers that have recently been reported for their high prognostic values. Previous studies have found a higher NLR at admission to be a significant independent predictor for poor functional outcomes in adult and pediatric patients with TBI (15, 16). On the other hand, MLR at admission has been shown to serve as a significant predictor for the development of acute traumatic intraparenchymal hemorrhage (tICH) (17).

NLR and MLR, as markers of systemic inflammation, vary with the progresses of brain contusion. In prior research, utilizing NLR and MLR upon admission may not provide a sufficiently accurate prediction of long-term prognosis after cerebral contusion. Rare study focused on the dynamic changes of NLR and MLR at different stages after a cerebral contusion and their association with the prognosis. Here, we aimed to further explore the dynamic change of NLR and MLR after cerebral contusion, and the association of NLR and MLR with the six-month prognosis in patients with cerebral contusion.

## Patients and methods

### Study design and patients

Hospitalized patients with the discharge diagnosis of cerebral contusions in two tertiary teaching hospitals in Guangdong, China from January 2016 to April 2020 (First and Second Affiliated Hospitals of Shantou University Medical College) were included in this retrospective study.

### Inclusion and exclusion criteria

Patients were included in the study if they met the following criteria: (1) baseline computed tomography (CT) showing head trauma intraparenchymal bleeding, plus (2) baseline and follow-up CT scans performed after a primary brain contusion. We excluded patients if they met any of the following criteria: (1) under 18 years old, (2) had undergone brain surgery prior to the follow-up CT scan, (3) baseline CT scan performed more than 6-hour or follow-up CT scan performed more than 48-hour after the onset of brain injury, (4) initial blood test not performed within 6-hour of the onset of brain injury, (5) had severe comorbidities such as neoplastic, cardiac, hepatic diseases, or renal diseases, or a history of neurological disease like head trauma, stroke, or brain tumor, or (6) had used immunomodulators or anticoagulants before the cerebral contusion.

(**Supplementary Figure 1**).

### Clinical data

Various demographic and clinical variables, such as sex, age, severity of injury mechanism, Glasgow Coma Scale (GCS) score, mean arterial pressure, hypertension, diabetes, and coagulation function, were acquired from the electronic medical record system of the hospitals. Venous blood samples were routinely collected for full blood count analysis immediately after hospital admission and at 24 hours and 1 week after cerebral contusion. Routine blood testing was conducted to determine leukocyte count (reference range of 3.5 – 9.5 × 10^9^ cells/L), monocyte count (reference range, 0.1 – 0.6 × 10^9^ cells/L), neutrophil count (reference range, 1.8 – 6.4 x 10^9^ cells/L), and lymphocyte count (reference range, 1.1 – 3.2 × 10^9^ cells/L). NLR was computed as the ratio of the absolute neutrophil count to the lymphocyte count; MLR was computed as the ratio of the absolute monocyte count to the lymphocytes count. When the activated partial thromboplastin time was greater than 36 seconds, the international normalized ratio was more than 1.2, or the platelet count exceeded 120 × 10^9^ platelets/L at admission, a patient was considered to have a coagulation disorder (18).

### Imaging data

An axial non-contrast CT image (5 mm slice thickness) of each participant was obtained using standardized procedures at their institutions. Three independent reviewers evaluated the CT images for each participant. Based on the CT images, the volume of the baseline and follow-up hematomas was calculated using semiautomated computer volumetric analysis (General Electric Company, Waukesha, WI, USA) (19). We manually selected the region of interest and then separated it from the surrounding environment using a Hounsfield unit threshold. Visually inspecting and manually adjusting the isolated regions to ensure that all three projections showed the hemorrhage. Hematoma volumes were automatically computed by summarizing adjacent voxels using a threshold (a fixed window of 110 and 50 HU) to distinguish hematomas from surrounding tissue. ICHs with multiple intraparenchymal hematomas (ICHs) in the contusion region were calculated on the basis of their total volume.

### Missing data

Several variables in the datasets contained missing data, including midline shift (missing data accounted for 26.4%), Cisterns compressed or absent (26.4%), coagulation function parameters (16.85%), severity of injury mechanism (7.79%), and hypertension (4.53%), diabetes (1.09%), and GCS level (0.62%). To preserve the sample size and limit the selection, missing values were imputed by chained equations for variables with fewer than 30% missing data points (20). It should be noted that only 202 out of 552 patients had 6-month GOS prognosis results. Therefore, the data of 552 patients was utilized to examine the relationship between NLR and MLR and tICH volume, and the data of 202 patients was used to investigate the association between NLR and MLR and 6-month adverse prognosis.

### Statistical analysis

The Kolmogorov-Smirnov test was used to assess the distribution mode of continuous variables. Normally distributed data was presented as mean ± standard deviation (SD) and analyzed using a t-test for comparison between two groups (with and without higher NLRs or higher MLRs). Non-normally distributed data was presented as medians (interquartile ranges, IQR) and analyzed using the Mann-Whitney U test for comparison between two groups. Categorical variables were presented as counts (percentages) and analyzed using the Chi-square test.

Univariate correlation analysis was conducted using Spearman Rho. NLR and LMR were individually analyzed using univariate logistic regression analysis, along with other variables (including sex, age, baseline GCS level, mean arterial pressure, hypertension, diabetes, subarachnoid hemorrhage, subdural hemorrhage, coagulopathy, location of contusion, tICH volume, midline shift and cisterns compressed or absent), to determine their ability to predict unfavorable outcomes after 6-month. Univariate logistic regression analysis was also used to identify potential risk factors for unfavorable outcomes after 6 months. Variables with a *P* < 0.20 in the univariate regression analyses were included in the multivariate regression model (21). The forward selection procedure was used to select independent risk factors for unfavorable 6-month outcomes, retaining variables with a *P* < 0.20. Additionally, other risk factors that have been reported in previous studies, such as hypertension and subarachnoid hemorrhage, were also included into the predictive models.

A receiver operating characteristic (ROC) curve was used to determine the optimal cut-off point of NLR or MLR for 6-month unfavorable outcome. All analyses were performed using the R-based statistical analysis platform: Extreme Smart Analysis (https://www.xsmartanalysis.com/) (22, 23). A two-tailed test of significance was used for all analyses.

## Results

### Patient characteristics

A total of 552 patients with cerebral contusions were included in the dataset. The mean age of the patients was 47.40 (SD, 17.09) with 73.19% males. Of the 273 (49.46%) patients, mild GCS levels were present at admission. Twenty-four (4.35%) patients died in the hospital, and 48 (23.76%) patients had an unfavorable outcome after 6 months. Of the 44 (8.01%) patients with subarachnoid hemorrhage (SAH), 368 (66.79%) had subdural hemorrhage (SDH). The mean NLR at admission (NLR_admission) was 13.48 (SD, 8.33), the 24-hour NLR (NLR_24h) was 9.18 (SD, 8.26), and the one-week NLR after cerebral contusion (NLR_1W) was 5.50 (SD, 4.09); the MLR at admission (MLR_admission) was 0.85 (SD, 0.54), the 24-hour MLR (MLR_24h) was 0.73 (SD, 0.48), and the one-week MLR (MLR_1W) was 0.62 (SD, 0.44).

### NLR and MLR dynamic change and association

**Figure 1:**
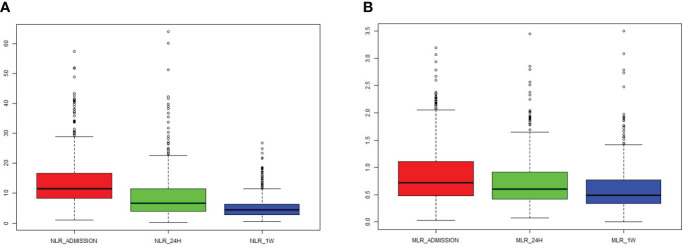
The dynamic change of NLR and MLR in the total patients with cerebral contusion. **(A)** The value of NLR at admission (within 6 hours after cerebral contusion), 24 hours and one week after cerebral contusion; **(B)** The value of MLR at admission (within 6 hours after cerebral contusion), 24 hours and one week after cerebral contusion.

Over the course of time, both NLR and MLR significantly decreased (**Figures 1A, B**). There was a correlation between NLR_admission and MLR_admission (R = 0.62, *P* < 0.001), NLR_24 h and MLR_24 h (R = 0.77, *P* < 0.001), and NLR_1W and MLR_1W after cerebral contusion (R = 0.75, *P* < 0.001), respectively (**Supplementary Figures 2A–C**).

### Correlation between NLR or MLR and the tICH volume

**Figure 2:**
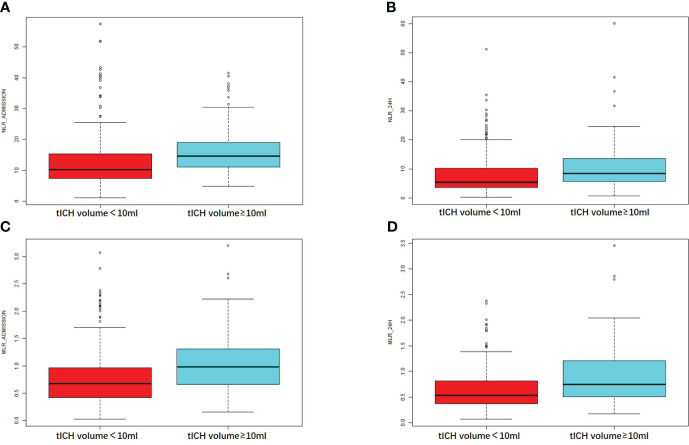
Comparison of the NLR or MLR in patients with different tICH volume. **(A, B)** The value of NLR at the admission hospital or at 24 hours in patients with or without tICH volume of no less than 10ml. **(C, D)** The value of MLR at the admission hospital or at 24 hours in patients with or without tICH volume of no less than 10ml.

NLR_admission and NLR_24h showed a positive correlation with tICH volume, according to the correlation analysis (R = 0.37, *P* < 0.001 and R = 0.25, *P* < 0.001) (**Figures 2A, B**). Meanwhile, A higher MLR_admission and a higher MLR_24h were associated with a greater volume of tICH (R = 0.33, *P* < 0.001and R = 0.24, *P* < 0.001, respectively) (**Figures 2C, D**).

### Correlation between MLR or MLR and 6-month unfavorable outcome

**Figure 3:**
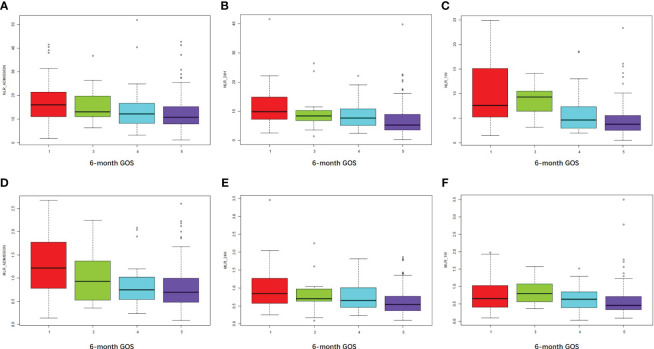
NLR and MLR were positively associated with an unfavorable outcome at 6 months. **(A–C)** NLR at admission, 24 hours, and one week after cerebral contusion in each GOS group. **(D–F)** MLR at admission, 24 hours, and one week after cerebral contusion in each GOS group.

Higher NLR_admission, NLR_24h, and NLR_1W were associated with an unfavorable prognosis as measured by 6-month GOS (R = 0.24, *P* < 0.001; R = 0.31, *P* < 0.001; R = 0.44, *P* < 0.001, respectively) (**Figures 3A–C**). Similarly, higher MLR_admission, MLR_24h, and MLR_1W were associated with a poorer 6-month outcome (R = 0.28, *P* < 0.001; R = 0.27, *P* < 0.001; R = 0.22, *P* = 0.004, respectively) (**Figures 3D–F**). Interestingly, neither NLR nor MLR were associated with in-hospital death (all the R values of NLR or MLR associations with in-hospital death were less than 0.2).

### NLR and MLR association with tICH volume on multivariate logistic regression

Univariate logistic regression analysis showed that NLR_admission or NLR_24h was associated with tICH volume (OR = 0.31, 95% CI, 0.12−0.50, *P* = 0.002; OR = 0.28, 95% CI, 0.06 - 0.50, *P* = 0.012, respectively). A significant association between NLR_admission and tICH volume remained after adjustment for age, baseline GCS level, mean arterial pressure, hypertension, presence of SAH, presence of SDH, coagulopathy, and location of contusion (aOR = 0.23, 95% CI, 0.04 - 0.41, *P* = 0.019) (**Supplementary Table 1**). Meanwhile, MLR_24h remained associated with tICH volume after adjustment for the covariates mentioned above (OR = 6.22, 95% CI, 2.39 - 10.05, *P* = 0.002) (**Supplementary Table 2**).

### Multivariate logistic regression analysis of NLR and MLR in relation to 6-month unfavorable prognosis

A univariate logistic regression analysis indicated that NLR_admission, NLR_24h, NLR_1W, MLR_admission, MLR_24h, and MLR_1W were associated with an unfavorable outcome at six months. After adjusting for age, baseline GCS level, hypertension, diabetes, neurosurgical treatment, presence of SAH, presence of SDH, presence of midline shift, presence of compressed or absent cisterns, and tICH volume, only NLR_1W and MLR_admission remained positively associated with a 6-month unfavorable outcome (1.17, 1.02 - 1.34, *P* = 0.021; 2.56, 0.91 - 7.17, *P* = 0.037, respectively) (**Supplementary Table 1**).

### NLR_1W and MLR_admission cut-off values for 6-month unfavorable prognosis

**Figure 4:**
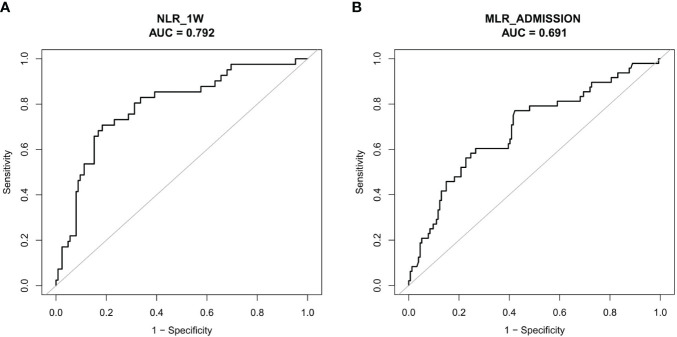
NLR_1W **(A)** and MLR_Admission **(B)** Receiver Operating Characteristic Curve Analysis.

In order to evaluate the overall discriminative ability of NLR_1W and MLR_admission for unfavorable outcomes, receiver operating characteristic curves were employed. The cut-off values for NLR_1W and MLR_admission with the best discrimination of unfavorable 6-month prognosis were 6.39 (70.73% sensitivity and 81.60% specificity) and 0.76 (77.08% sensitivity and 57.79% specificity), respectively (**Figures 4A, B**).

The patients with high NLR_1W (>6.39) were not significantly different in the proportion of male patients (72.70% *vs* 75.63%, *P* < 0.531), but had an older age (53.03 ± 18.90 *vs* 45.86 ± 15.91 years, *P* < 0.001) and a high-proportioned severe GCS level (48.74% *vs* 23.12%, *P* < 0.001), and a high-proportioned in-hospital death (12.61% *vs* 1.11%, *P* < 0.001) (**Table 1**). In contrast, the patients with high MLR_admission (>0.76) were not significantly different in age (47.02 ± 18.60 *vs* 47.71 ± 15.77, *P* < 0.001), but had a high-proportioned male patient (82.00% *vs* 65.89%, *P* < 0.001), a high-proportioned severe GCS level (34.00% *vs* 24.17%, *P* = 0.020) and a high-proportioned in-hospital death (8.00% *vs* 1.32%, *P* < 0.001) (**Table 2**).

**Table 1:** Comparisons of demographics between the NLR_1W groups.

Variables	≤ 6.39 (n=359)	> 6.39 (n=119)	Standardize diff.	*P*-value
Male	261 (72.70)	90 (75.63)	0.07 (-0.14, 0.27)	0.531
Age, years	45.86 ± 15.91	53.03 ± 18.90	0.41 (0.20, 0.62)	<0.001
MAP, mmHg	99.14 ± 18.04	103.81 ± 17.01	0.27 (0.06, 0.47)	0.013
**GCS level**			0.66 (0.44, 0.87)	<0.001
Mild	200 (55.71)	32 (26.89)		
Moderate	76 (21.17)	29 (24.37)		
Severe	83 (23.12)	58 (48.74)		
**Injury mechanism**			0.14 (-0.08, 0.35)	0.485
Mild	82 (24.85)	21 (19.27)		
Moderate	13 (3.94)	5 (4.59)		
Severe	235 (71.21)	83 (76.15)		
Hypertension	21 (6.05)	23 (19.83)	0.42 (0.21, 0.63)	0.001
Diabetes	15 (4.24)	8 (6.72)	0.11 (-0.10, 0.32)	0.275
Nuerosurgery treatment	71 (19.78)	42 (35.29)	0.35 (0.14, 0.56)	<0.001
Intraventricular hemorrhage	21 (5.88)	19 (15.97)	0.33 (0.12, 0.54)	<0.001
Subarachnoid hemorrhage	261 (73.11)	95 (79.83)	0.16 (-0.05, 0.37)	0.144
Subdural hemorrhage	233 (65.08)	86 (72.27)	0.16 (-0.05, 0.36)	0.149
Extradural hemorrhage	87 (24.37)	27 (22.69)	0.04 (-0.17, 0.25)	0.71
Coagulopathy	21 (7.22)	13 (13.13)	0.20 (-0.03, 0.43)	0.072
**Location of contusion**			0.12 (-0.08, 0.33)	0.828
Frontal	162 (45.13)	52 (43.70)		
Temporal	156 (43.45)	52 (43.70)		
Parietal	19 (5.29)	5 (4.20)		
Occipital	7 (1.95)	2 (1.68)		
Basal ganglia, brainstem, or cerebellum	15 (4.18)	8 (6.72)		
tICH volume, ml	7.87 ± 14.49	14.50 ± 19.11	0.39 (0.16, 0.62)	<0.001
In-hospital death	4 (1.11)	15 (12.61)	0.47 (0.26, 0.68)	<0.001
**6-month unfavorable prognosis**	12 (10.53)	29 (55.77)	1.10 (0.75, 1.44)	<0.001

tICH, acute traumatic intraparenchymal hematoma, referring to the largest volume of parenchymatous hematoma within 48 hours after cerebral contusion as measured by baseline CT or follow-up CT.

**Table 2:** Comparisons of demographics between the MLR_admission groups.

Variables	≤ 0.76 (n=302)	> 0.76 (n=250)	Standardize diff.	*P*-value
Male	199 (65.89)	205 (82.00)	0.37 (0.20, 0.54)	<0.001
Age, years	47.71 ± 15.77	47.02 ± 18.60	0.04 (-0.13, 0.21)	0.639
MAP, mmHg	99.15 ± 17.43	101.26 ± 18.29	0.12 (-0.05, 0.29)	0.166
**GCS level**			0.24 (0.07, 0.41)	0. 020
Mild	164 (54.30)	109 (43.60)		
Moderate	65 (21.52)	56 (22.40)		
Severe	73 (24.17)	85 (34.00)		
**Injury mechanism**			0.14 (-0.04, 0.31)	0.311
Mild	59 (21.15)	56 (24.45)		
Moderate	8 (2.87)	11 (4.80)		
Severe	212 (75.99)	162 (70.74)		
Hypertension	23 (8.01)	26 (10.83)	0.10 (-0.07, 0.27)	0.267
Diabetes	13 (4.36)	11 (4.44)	0.00 (-0.16, 0.17)	0.967
Neurosurgery treatment	49 (16.23)	74 (29.60)	0.32 (0.15, 0.49)	<0.001
Intraventricular hemorrhage	19 (6.33)	25 (10.04)	0.14 (-0.03, 0.30)	0.111
Subarachnoid hemorrhage	211 (70.10)	196 (78.71)	0.20 (0.03, 0.37)	0.022
Subdural hemorrhage	182 (60.47)	186 (74.40)	0.30 (0.13, 0.47)	<0.001
Extradural hemorrhage	71 (23.59)	64 (25.70)	0.05 (-0.12, 0.22)	0.566
Coagulopathy	18 (7.44)	24 (11.06)	0.13 (-0.06, 0.31)	0.179
**Location of contusion**			0.10 (-0.07, 0.26)	0.870
Frontal	133 (44.04)	119 (47.60)		
Temporal	136 (45.03)	101 (40.40)		
Parietal	14 (4.64)	13 (5.20)		
Occipital	5 (1.66)	5 (2.00)		
Basal ganglia, brainstem, or cerebellum	14 (4.64)	12 (4.80)		
tICH volume, ml	6.16 ± 12.16	12.16 ± 17.71	0.40 (0.21, 0.58)	<0.001
In-hospital death	4 (1.32)	20 (8.00)	0 32 (0.15, 0.49)	<0.001
**6-month unfavorable prognosis**	11 (11.00)	37 (36.27)	0.62 (0.34, 0.91)	<0.001

tICH, acute traumatic intraparenchymal hematoma, referring to the largest volume of parenchymatous hematoma within 48 hours after cerebral contusion as measured by baseline CT or follow-up CT.

## Discussion

This retrospective study revealed a significant association between NLR and MLR at multiple time intervals after a brain contusion. Early inflammatory markers like NLR_admission, MLR_admission and MLR_24h were independently associated with the volume of cerebral parenchymal hematomas. Moreover, later inflammation indicators such as NLR_1W could independently be correlated with a poor long-term prognosis and had certain clinical predictive value. As time passes following a brain contusion, NLR has a greater capacity to predict a poor prognosis, while MLR has a reduced capacity to make such a distinction. NLR_1W and MLR_admission exhibited optimal discrimination for a 6-month unfavorable outcome after a brain contusion, respectively. These inflammatory indicators can help surgeons anticipate a long-term prognosis in patients with brain contusions and enhance the stratified management of these individuals.

TBI affects millions of individuals globally each year (22). The pathophysiology of TBI is a highly complex process that involves both the primary brain injury caused by external trauma (21) and the secondary injury that occurs within minutes of the primary one and can persist for several days thereafter (22). This secondary injury is believed to result from a series of cellular and molecular events and processes, including neuroinflammation, excitatory neurotoxicity, lipid peroxidation, edema, and mitochondrial dysfunction (23–26). Neuroinflammation has been identified as a key factor in the development of TBI, leading to investigation of various immune responses, both pro-inflammatory and anti-inflammatory (27). Following a traumatic brain injury, the body initiates a systemic immune response that leads to significant alterations in various immune cells and inflammatory markers, including NLR and MLR (26). Moreover, accumulating evidence suggests a potential association between TBI and the development of chronic traumatic encephalopathy (CTE), a neurodegenerative condition characterized by the presence of abnormal hyperphosphorylated tau (p-tau) accumulating in neurons and astroglia, located around small blood vessels at the depths of cortical sulci and in an irregular pattern (28). Thus, elevated NLR and MLR levels following TBI or brain contusion could indicate heightened neuroinflammation and systemic inflammatory response, potentially leading to a more severe or prolonged CTE, and resulting in an unfavorable long-term prognosis.

Consistent with previous findings (15, 17), our study demonstrated the predictive ability of NLR_1W and MLR-admission for 6-month poor prognosis after brain contusion and identified their respective cutoff values. Surgeons can utilize this information in clinical practice to make preliminary assessments of the long-term prognosis of patients with brain contusions. This, in turn, will contribute to enhancing the treatment and management of patients who are anticipated to have a poor prognosis in the early stages of brain contusions.

The results section revealed the optimal cutoff values for predicting 6-month unfavorable outcomes for NLR_1W and MLR_admission to be 6.39 and 0.76, respectively. Surgeons can utilize these thresholds to quickly assess the prognostic risks for patients with brain contusions and make informed decisions regarding follow-up treatment and management. Specifically, patients with mild or moderate brain contusions who exhibit an NLR_1W of > 6.39 or an MLR_admission of > 0.76 are at a higher risk of experiencing a poor prognosis. Early identification of these patients allows attending physicians to implement more proactive treatment strategies and tailored management plans, thereby, improving outcomes for individuals with brain contusions. However, it is important to note that while these inflammatory indicators are valuable in guiding surgeons during the assessment of patient prognosis, they should not be regarded as a substitute for comprehensive evaluations conducted by surgeons. Given that both NLR and MLR can be obtained through cost-efficient and readily available white blood cell differentials, they could represent promising parameters for risk stratification in patients with cerebral contusion, and could complement established biomarkers in the diagnosis and treatment of affected patients.

NLR represents a combination indicator of total neutrophil and lymphocyte counts in the peripheral blood and has been reported to have the potential to predict the acute-phase progression of brain parenchymal hematomas or long-term prognosis in patients with cerebral contusion (29, 30). Consistently, our results exhibited an apparent positive association between early inflammation indices (NLR_admission and NLR_24 h) and acute tICH volume. However, numerous studies have only focused on the admission or baseline NLR index to predict long-term outcomes. Neutrophils are the primary cells responsible for innate immunity following a brain contusion. Within 6 to 24 hours, perivascular neutrophils migrate into the parenchyma (31, 32). In the course of the illness, their numbers undergo considerable fluctuations over the course of a few days, as they have a short lifespan. In this way, dynamic measurements of NLR may serve as a better predictor than single measurements of NLR.

Recently, Ehsan Alimohammadi et al., reported that the dynamic of NLR can be used to predict the clinical outcome of children with TBI (33). As far as we know, adults with cerebral contusions have never had dynamic NLR measurements. Our results demonstrate that the NLR value decreases gradually over time, but the NLR in patients with a poor prognosis remains higher than that of patients with a good prognosis at the same time point. Furthermore, the ability of a single NLR to predict a 6-month poor prognosis is becomes more powerful over time, with NLR_1W having the greatest discrimination for a 6-month poor prognosis compared to NLR_admission and NLR_24h. Thus, dynamic detection of NLR is considerably important in forecasting the long-term prognosis of patients with brain contusion.

Similarly, MLR is another important inflammatory marker used to predict stroke and spontaneous ICH prognosis (34, 35). A significant independent association was found between MLR and hemorrhagic transformation in patients with acute ischemic stroke, as well as neurological disabilities and brain atrophy in patients with multiple sclerosis (36, 37). In this study, we found an apparent association between MLR at admission and acute tICH volume. Our recent study demonstrated that MLR at admission can predict the progression of acute traumatic parenchymal hematomas after cerebral contusions (17). While the predictive value of dynamic MLR for long-term prognosis in cerebral contusion has not been fully explored. MLR decreases gradually over time after cerebral contusion with one week. In patients with a 6-month unfavorable outcome, MLR remained higher than that in patients with a 6-month good outcome at the same time point (**Supplementary Figure 3**).

In our findings, in general, NLR exhibited a better predictive value than MLR for a 6-month unfavorable prognosis. Additionally, the MLR in patients with favorable outcomes remained stable from admission to one week after cerebral contusion, while the count of neutrophils had a significant decrease in these patients with unfavorable outcomes over time (**Supplementary Figure 3**). We theorize that in patients with a favorable prognosis, after the initial brain contusion, there may not be new damage points, i.e., not enough damage-associated molecular patterns (DAMP), to further stimulate the proliferation and activation of monocytes in peripheral blood and their infiltration of monocytes into brain tissue (38). On the other hand, in patients with an unfavorable outcome, the monocyte count seemingly has an apparent increase and gradual decrease over time. In comparison to monocytes, neutrophils have a greater presence in peripheral blood leukocytes and are more sensitive as an indicator of inflammation (39). Neutrophils constitute 60 to 70% of peripheral white blood cells, while monocytes/macrophages accounting for 3 to 8% (40). During the initial stage of brain contusion, besides the activation of resident microglia and astrocytes in the brain, neutrophils are the innate immune cells that initiate the response initially, increasing in number, and migrating to the lesion area and its surroundings. Following that, monocytes/macrophages start to infiltrate into the damaged brain area, serving the purpose of eliminating broken cell pieces and amplifying the inflammatory response, and helping to mend the harmed lesion in the late stage (41). Hence, the count of neutrophils in peripheral blood appears to be more responsive to cerebral contusion compared to the count of monocytes. This may explain why the NLR demonstrates a higher predictive accuracy for long-term prognosis than the MLR.

It is also worth noting that, as time passes, the discrimination of NLR from unfavorable prognosis improves, while the discrimination of MLR for unfavorable prognosis weakens. NLR and MLR are two simple markers of inflammation, determined by the number of cells in the peripheral blood. First, as previously stated, the number of neutrophils is more sensitive to continued deterioration after cerebral contusion than the number of monocytes in the peripheral blood. Second, this difference can be attributed to the distinct functional characteristics of the two innate immune cells. Following a brain contusion, neutrophils rapidly accumulate at the damaged site and isolate or eliminate broken cell fragments via phagocytosis or a trap net created by cellular self-destruction (42, 43). Neutrophils are unable to repeat these activities and have a short half-life; therefore, their involvement in the inflammatory response is dependent upon the number of cells present. Monocytes undergo certain levels of proliferation and activation when stimulated by cytokines at the start of brain injury. These cells travel to the affected area and then differentiate into macrophages, which are mainly responsible for repeatedly clearing away debris, as well as releasing cytokines that will aggravate local inflammation at the site of injury and assist in the adaptive immune response process that follows (44, 45). As the injury progresses, the macrophages can be transformed into anti-inflammatory macrophages to help with repairing the damage (46). Thus, the immune functions of monocytes/macrophages are not only determined by their number, but also by their multi-functionality. This means that, at later stages, the number of monocytes present may not be a reliable indicator of the severity of brain contusion.

Compared to neutrophils and monocytes, the role of lymphocytes in long-term outcome after cerebral contusion remains unclear. Consistent with previous studies, a lower lymphocyte count was observed among patients with an unfavorable prognosis, which was likely attributed to a decrease in T lymphocytes (47, 48). Patients with traumatic brain injury and reduced T lymphocyte counts suffered significantly worse neurological outcomes and had an increased incidence of pulmonary infection (49). Induction of lymphocytes after cerebral contusion may therefore improve functional outcomes. The possible role of T lymphocytes in cerebral contusion may be complicated by the presence of multiple subtypes and bidirectional immunomodulatory functions. Further study is required to determine whether T lymphocytes play a role in the prognosis of victims with cerebral contusion.

Interpreting the current findings requires consideration of several limitations. Firstly, the retrospective design of the study may introduce selection and information biases. To mitigate these biases, efforts were to include patients from two tertiary comprehensive hospitals known for their high-quality medical records. Additionally, appropriate statistical analyses, such as multivariate regression, were employed to isolate the effect of the exposure variable on the outcome variable while accounting for other potentially influential variables.

Secondly, a significant number of patients in the retrospective dataset lacked long-term follow-up data for prognosis assessment. The analysis of long-term prognosis was therefore conducted on a small sample size of 202 cases. Further validation of the predictive value of NLR_1W and MLR_admission for unfavorable outcomes after 6-month may require a prospective multicenter study with a larger cohort.

Thirdly, the dynamic analysis of NLR and MLR in our retrospective datasets was limited to only three time points. Conducting more rigorous prospective studies with multiple time points would be crucial in order to gain a more comprehensive understanding of the dynamic changes in these markers following brain contusion.

Fourthly, it is important to consider the potential variations in NLR and MLR measurements between the laboratories of the two participating hospitals. Addressing potential sources of variability in the measurements of NLR and MLR would enhance the validity and reliability of the data.

Finally, routine blood analysis provided only a broad measure of monocytes and lymphocytes, without considering subsets such as nonclassical monocytes and T lymphocyte that have been implicated in neurological deterioration after cerebral contusion (47, 50). Conducting a more comprehensive examination of white blood cell subpopulations in peripheral blood samples from individuals with brain contusion could help identify specific immune cell subpopulations that contribute to unfavorable outcomes, thereby providing insights for the development of novel diagnostic and management targets in the future.

## Conclusions

In conclusion, NLR and MLR at multiple time intervals after a cerebral contusion are significantly associated. As time passes following a brain contusion, NLR has a greater capacity to discriminate a poor six-month prognosis, while MLR has a reduced capacity to make such a distinction. A high NLR_1W (>6.39) and a high MLR_admission (>0.76) likely indicate an unfavorable 6-month prognosis in patients with cerebral contusion. These indicators can help surgeons anticipate a poor prognosis in patients with brain contusions and enhance the stratified management of these individuals.

## Data availability statement

The original contributions presented in the study are included in the article/**Supplementary Material**, further inquiries can be directed to the corresponding author/s.

## Ethics statement

The studies involving humans were approved by the ethics committee at the First Affiliated Hospital of Shantou University Medical College, and the Second Affiliated Hospital of Shantou University Medical College has approved this study (No.: 2020-042). The studies were conducted in accordance with the local legislation and institutional requirements. Written informed consent for participation was not required from the participants or the participants’ legal guardians/next of kin because the retrospective nature of the study.

## Author contributions

DZ: Investigation, Writing – original draft. DZZ: Data curation, Writing – review & editing, Formal analysis, Investigation, Methodology. TL: Data curation, Writing – review & editing, Formal analysis, Investigation, Methodology. XL: Formal analysis, Writing – review & editing. ZZ: Formal analysis, Methodology, Writing – review & editing. LZ: Formal analysis, Methodology, Writing – review & editing. FT: Data curation, Resources, Writing – review & editing. XC: Investigation, Methodology, Writing – review & editing. KL: Supervision, Writing – review & editing. WC: Conceptualization, Funding acquisition, Supervision, Writing – review & editing, Resources. JS: Conceptualization, Funding acquisition, Methodology, Software, Supervision, Writing – review & editing.

## Supplementary material

Supplementary Figure 1 The Flowchart of the Patient Selection Process, including Inclusion and Exclusion Criteria.

Supplementary Figure 2 The association between the NLR and MLR at admission **(A)**, at 24 hours **(B)**, and at one week **(C)** after a cerebral contusion, respectively.

Supplementary Figure 3 The dynamic change of NLR **(A)**, MLR **(B)**, neutrophils **(C)**, monocytes **(D)** and lymphocytes **(E)** in patients with or without an unfavorable 6-month prognosis. In these figures, “o” stands for a favorable 6-month prognosis, and “1” refers to an unfavorable 6-month prognosis.
