# Supplementary material for: An analysis of neutrophil-to-lymphocyte ratios and monocyte-to-lymphocyte ratios with six-month prognosis after cerebral contusions
Source: Front Immunol. 2024 Mar 12;15:1336862. doi: 10.3389/fimmu.2024.1336862 (PMC10967015; doi:10.3389/fimmu.2024.1336862)
Supplement: Supplementary file 1 [file Table_1.docx]

**Supplementary Table 1:** Logistic regression NLR_admission and NLR_24h model for tICH volume

| **Variables** | **NLR_admission model for tICH volume** | | **NLR_24h model for tICH volume** | |
| --- | --- | --- | --- | --- |
|  | **Odds Ratio (95% CI)** | **P Value** | **Odds Ratio (95% CI)** | **P Value** |
| Age (>65 years vs ≤ 65 years) | 3.94 (-0.51, 8.39) | 0.084 | 3.18 (-1.90, 8.26) | 0.220 |
| Level on Glasgow Coma Scale score, no. (%) |  |  |  |  |
| Mild (13–15 points) | 1 [Reference] | 1 [Reference] | 1 [Reference] | 1 [Reference] |
| Moderate (9–12 points) | 4.21 (0.18, 8.23) | 0.041 | 5.61 (1.02, 10.19) | 0.017 |
| Severe (3–8 points) | 4.84 (1.23, 8.44) | 0.009 | 4.33 (0.10, 8.55) | 0.046 |
| Mean arterial pressure, median, mHg | 0.07 (-0.03, 0.17) | 0.147 | 0.07 (-0.04, 0.18) | 0.235 |
| Hypertension (Yes vs No) | -0.88 (-6.92, 5.16) | 0.775 | 0.23 (-6.63, 7.09) | 0.948 |
| Subarachnoid hemorrhage (Yes vs No) | 1.29 (-2.39, 4.98) | 0.491 | 0.96 (-3.42, 5.34) | 0.668 |
| Subdural hemorrhage (Yes vs No) | 4.29 (0.69, 7.90) | 0.020 | 5.75 (1.61, 9.90) | 0.007 |
| Coagulopathy (Yes vs No) | 0.88 (-4.17, 5.94) | 0.733 | 1.16 (-4.30, 7.53) | 0.593 |
| Location of contusion |  |  |  |  |
| Frontal | 1 [Reference] | 1 [Reference] | 1 [Reference] | 1 [Reference] |
| Parietal | -1.56 (-4.80, 1.69) | 0.347 | -2.26 (-6.00, 1.49) | 0.239 |
| Others | -3.57 (-8.43, 1.28) | 0.150 | -4.87 (-10.49, 0.75) | 0.090 |
| **NLR_admission/ 24h** | **0.23 (0.04, 0.41)** | **0.019** | **0.23 (-0.02, 0.47)** | **0.068** |

tICH, acute traumatic intraparenchymal hematoma, referring to the largest volume of parenchymatous hematoma

within 48 hours after cerebral contusion as measured by baseline CT or follow-up CT.
