# Supplementary material for: An analysis of neutrophil-to-lymphocyte ratios and monocyte-to-lymphocyte ratios with six-month prognosis after cerebral contusions
Source: Front Immunol. 2024 Mar 12;15:1336862. doi: 10.3389/fimmu.2024.1336862 (PMC10967015; doi:10.3389/fimmu.2024.1336862)
Supplement: Supplementary file 2 [file Table_2.docx]

**Supplementary Table 2:** Logistic regression MLR_admission and MLR_24h model for tICH volume

| **Variables** | **MLR_admission model for tICH volume** | | **MLR_24h model for tICH volume** | |
| --- | --- | --- | --- | --- |
|  | **Odds Ratio (95% CI)** | **P Value** | **Odds Ratio (95% CI)** | **P Value** |
| Age (>65 years vs ≤ 65 years) | 4.17 (-0.30, 8.64) | 0.068 | 2.75 (-2.28, 7.78) | 0.284 |
| Level on Glasgow Coma Scale score, no. (%) |  |  |  |  |
| Mild (13–15 points) | 1 [Reference] | 1 [Reference] | 1 [Reference] | 1 [Reference] |
| Moderate (9–12 points) | 4.17 (0.09, 8.25) | 0.046 | 5.24 (0.70, 9.77) | 0.024 |
| Severe (3–8 points) | 4.75 (1.11, 8.39) | 0.011 | 3.98 (-0.16, 8.12) | 0.061 |
| Mean arterial pressure, median, mHg | 0.07 (-0.03, 0.17) | 0.152 | 0.05 (-0.06, 0.16) | 0.341 |
| Hypertension (Yes vs No) | -1.07 (-7.13, 5.00) | 0.731 | 0.16 (-6.61, 6.93) | 0.963 |
| Subarachnoid hemorrhage (Yes vs No) | 1.58 (-2.11, 5.28) | 0.403 | 0.87 (-3.46, 5.19) | 0.695 |
| Subdural hemorrhage (Yes vs No) | 4.75 (1.17, 8.33) | 0.010 | 6.21 (2.11, 10.31) | 0.003 |
| Coagulopathy (Yes vs No) | 0.95 (-4.12, 6.03) | 0.713 | 1.51 (-4.34, 7.35) | 0.614 |
| Location of contusion |  |  |  |  |
| Frontal | 1 [Reference] | 1 [Reference] | 1 [Reference] | 1 [Reference] |
| Parietal | -1.62 (-4.87, 1.64) | 0.332 | -1.95 (-5.66, 1.75) | 0.302 |
| Others | -3.68 (-8.55, 1.19) | 0.139 | -4.16 (-9.72, 1.40) | 0.143 |
| **MLR_admission/ 24h** | **4.17 (-0.29, 5.28)** | **0.079** | **6.22 (2.39, 10.05)** | **0.002** |

tICH, acute traumatic intraparenchymal hematoma, referring to the largest volume of parenchymatous hematoma

within 48 hours after cerebral contusion as measured by baseline CT or follow-up CT.
