# Supplementary material for: An analysis of neutrophil-to-lymphocyte ratios and monocyte-to-lymphocyte ratios with six-month prognosis after cerebral contusions
Source: Front Immunol. 2024 Mar 12;15:1336862. doi: 10.3389/fimmu.2024.1336862 (PMC10967015; doi:10.3389/fimmu.2024.1336862)
Supplement: Supplementary file 3 [file Table_3.docx]

**Supplementary Table 3:** Logistic regression NLR_1W and MLR_admission model for 6-month unfavorable prognosis

| **Variables** | **NLR_1W model for 6-month unfavorable prognosis** | | **MLR_admission model for 6-month unfavorable prognosis** | |
| --- | --- | --- | --- | --- |
|  | **Odds Ratio (95% CI)** | **P Value** | **Odds Ratio (95% CI)** | **P Value** |
| Age (>65 years vs ≤ 65 years) | 6.49 (0.93, 45.38) | 0.060 | 10.18 (1.78, 58.27) | 0.009 |
| Level on Glasgow Coma Scale score, no. (%) |  |  |  |  |
| Mild (13–15 points) | 1 [Reference] | 1 [Reference] | 1 [Reference] | 1 [Reference] |
| Moderate (9–12 points) | 3.13 (0.39, 24.89) | 0.280 | 2.72 (0.52, 14.29) | 0.238 |
| Severe (3–8 points) | 9.42 (1.47, 60.43) | 0.018 | 7.00 (1.59, 30.82) | 0.010 |
| Hypertension (Yes vs No) | 0.46 (0.50, 4.39) | 0.500 | 0.83 (0.10, 6.84) | 0.859 |
| Neurosurgical treatment (Yes vs No) | 2.11 (0.37, 12.10) | 0.402 | 2.47 (0.56, 10.91) | 0.233 |
| Mild shift (Yes vs No) | 9.85 (1.10, 88.48) | 0.041 | 4.45 (0.66, 30.10) | 0.126 |
| Cisterns compressed or absent (Yes vs No) | 2.57 (0.47, 14.20) | 0.278 | 2.91 (0.63, 13.34) | 0.169 |
| tICH volume (＞10 ml vs ≤10 ml) | 0.96 (0.47, 14.20) | 0.278 | 1.37 (0.37, 5.09) | 0.638 |
| **NLR_1W/MLR_admission** | **1.17 (1.02, 1.34)** | **0.021** | **2.56 (0.91, 7.17)** | **0.037** |

tICH, acute traumatic intraparenchymal hematoma, referring to the largest volume of parenchymatous hematoma

within 48 hours after cerebral contusion as measured by baseline CT or follow-up CT.
