# Supplementary figures and images for: An analysis of neutrophil-to-lymphocyte ratios and monocyte-to-lymphocyte ratios with six-month prognosis after cerebral contusions
Source: Front Immunol. 2024 Mar 12;15:1336862. doi: 10.3389/fimmu.2024.1336862 (PMC10967015; doi:10.3389/fimmu.2024.1336862)

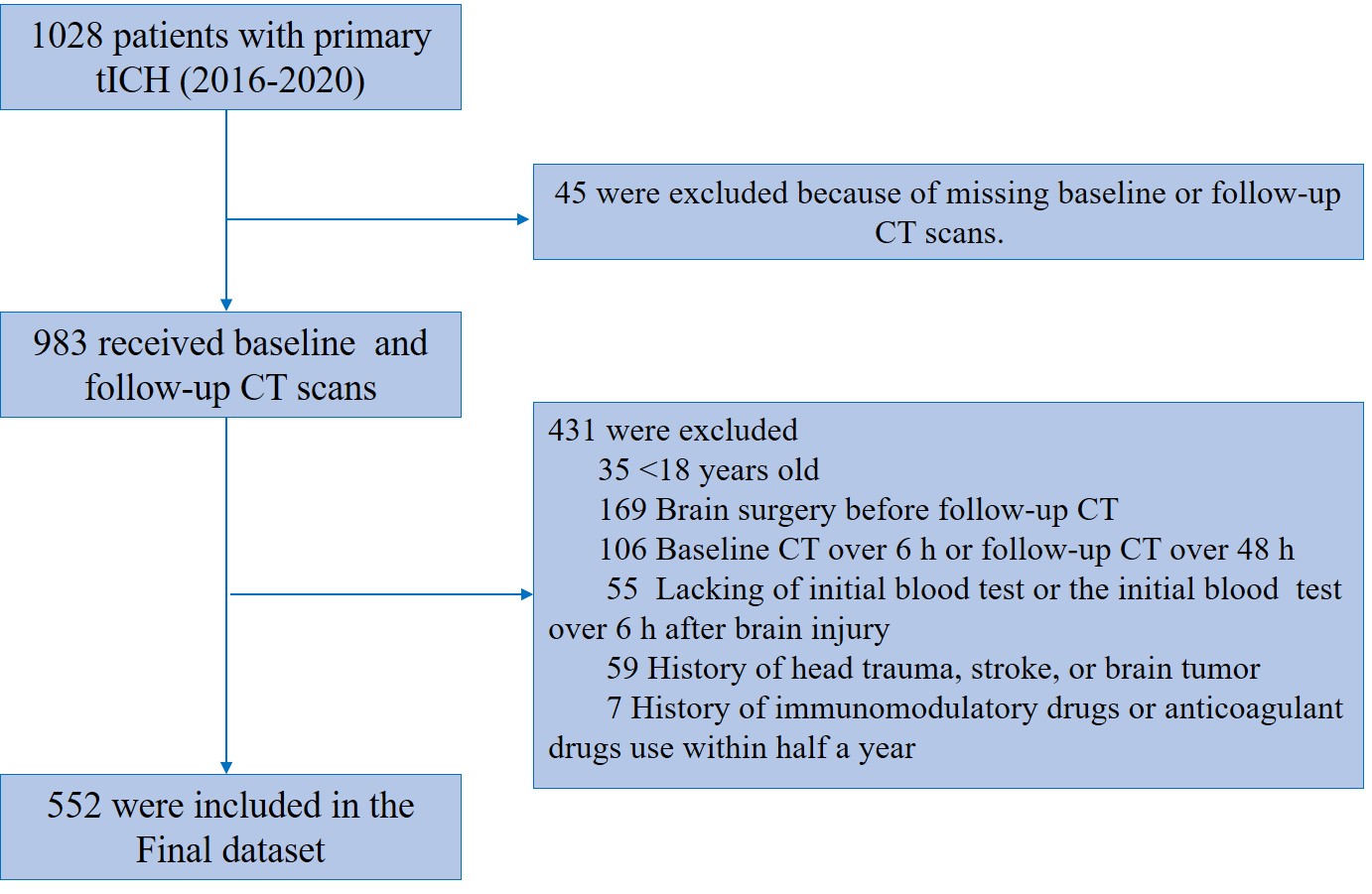

Supplement: Supplementary Figure 1 — The Flowchart of the Patient Selection Process, including Inclusion and Exclusion Criteria. [file Image_1.jpeg]

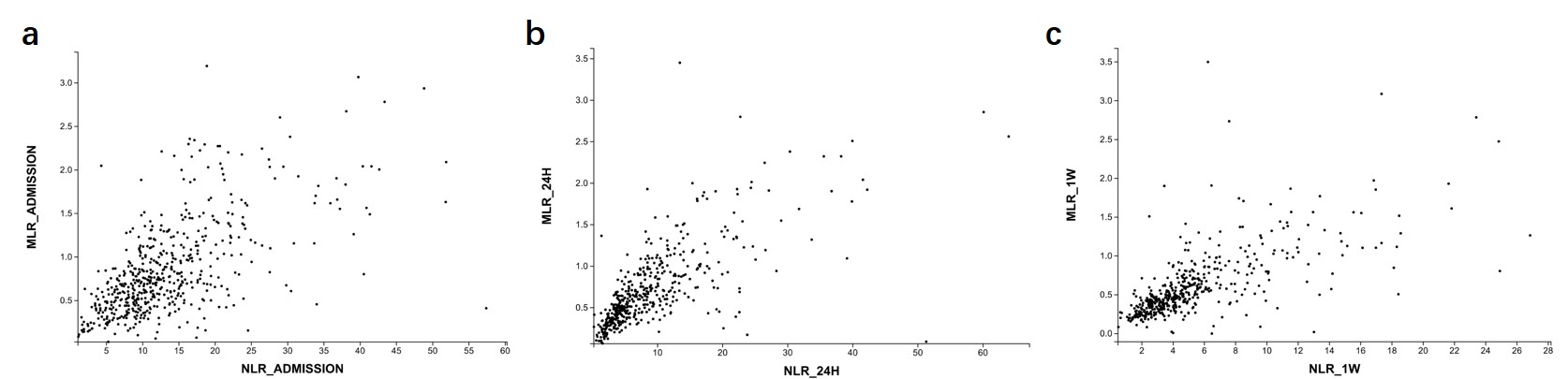

Supplement: Supplementary Figure 2 — The association between the NLR and MLR at admission (A), at 24 hours (B), and at one week (C) after a cerebral contusion, respectively. [file Image_2.jpeg]

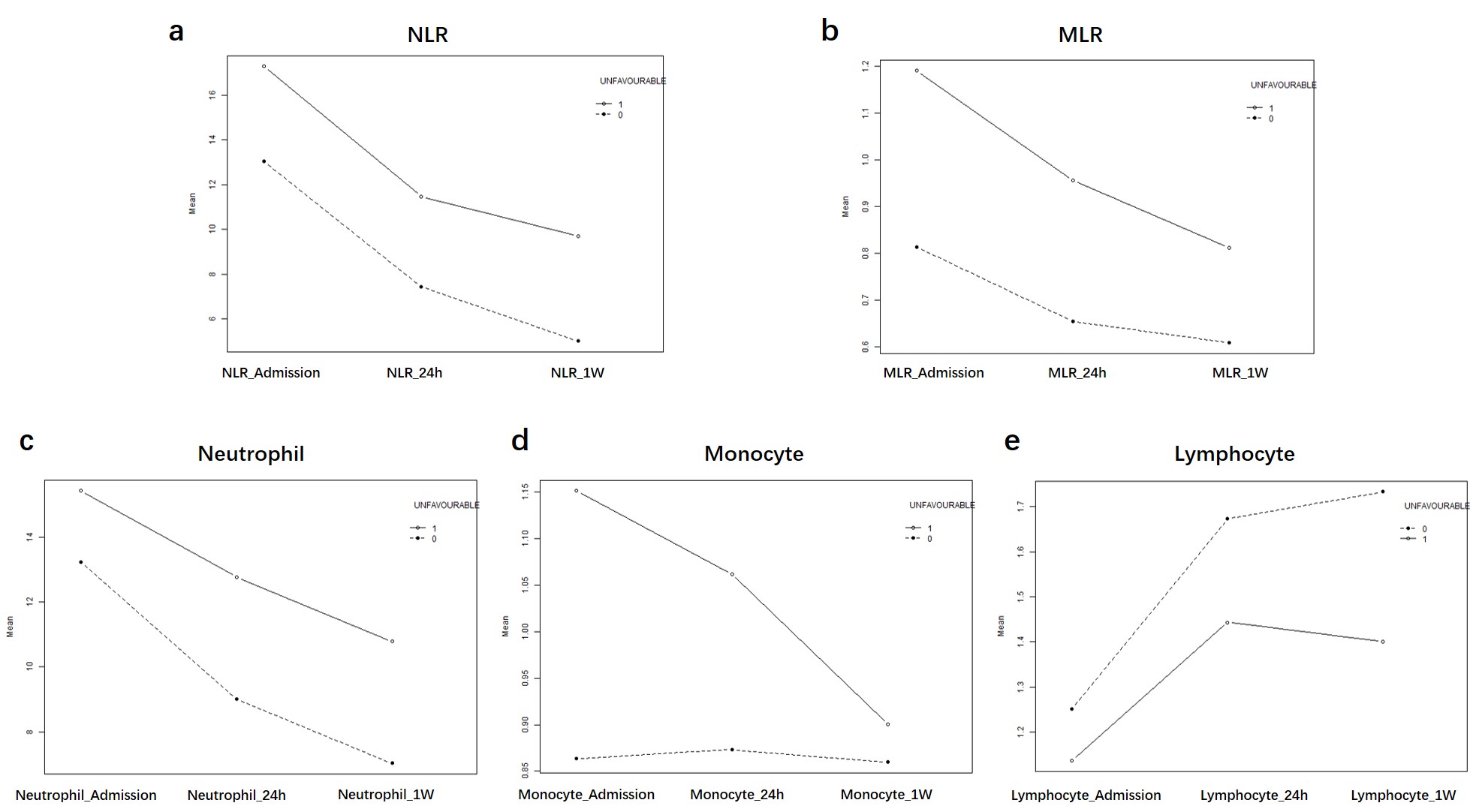

Supplement: Supplementary Figure 3 — The dynamic change of NLR (A), MLR (B), neutrophils (C), monocytes (D) and lymphocytes (E) in patients with or without an unfavorable 6-month prognosis. In these figures, “o” stands for a favorable 6-month prognosis, and “1” refers to an unfavorable 6-month prognosis. [file Image_3.jpeg]
